# Breaking Stigmas: innovative medical training in mental health through community-based partnership

**DOI:** 10.1192/j.eurpsy.2026.10621

**Published:** 2026-07-31

**Authors:** A. D. A. Pereira

**Affiliations:** Mental Health, Unifenas Medical Course, Belo Horizonte, Brazil

## Abstract

**Introduction:**

Stigma toward people with mental disorders remains a global challenge, negatively affecting healthcare quality and discouraging psychiatry as a career choice. Evidence suggests that well-designed educational experiences can reduce prejudice and foster greater interest in psychiatry.

**Objectives:**

To evaluate the impact of innovative educational strategies in mental health, developed through a partnership between the UNIFENAS-BH medical school and the Psychosocial Care Network (PCN) of Belo Horizonte, on medical students’ stigma toward people with mental disorders.

**Methods:**

A quasi-experimental pre–post design was applied, including 156 medical students from different stages of training (1st, 4th and 6th year). Three curricular strategies were assessed: Community Medical Practice (CMP), Neuropsychiatry Discipline (ND), and Internship in Mental Health Emergencies (IMHE). Stigma was measured with the Brazilian version of the Attribution Questionnaire (AQ-26B). In addition, a six-year longitudinal cohort of 76 students was analyzed.

**Results:**

A significant reduction in stigma was found across all curricular interventions combined (p < 0.001). The CMP showed the strongest impact, with significant decreases in Segregation (p = 0.004), Withdrawal (p < 0.001), Fear (p = 0.010) and Coercion to treatment (p = 0.004). The IMHE also produced positive effects, especially reducing Fear (p = 0.001). In contrast, the ND showed limited effect and a slight increase in Coercion (p = 0.006).Longitudinal analysis confirmed a significant inversion in stigma levels: of 24 students who changed scores, 22 reduced their stigma degree between 2013 and 2019 (p < 0.05).Graduate evaluations indicated progressive improvement in mental health training after curricular reforms.

**Conclusions:**

The integration of community-based mental health services into medical education and the adoption of active learning methodologies, centered on direct interaction with service users, effectively reduced stigma among medical students. These findings highlight the importance of longitudinal, practical, and human-centered curricular strategies to strengthen medical training in mental health.

**Disclosure of Interest:**

None Declared

